# Insomnia, pruritus, and constipation in hemodialysis patients: a cross-sectional study

**DOI:** 10.3389/fphys.2025.1637989

**Published:** 2025-08-04

**Authors:** Jing Zhu, Bifei Wang

**Affiliations:** Department of Nephrology, Shenzhen Luohu People’s Hospital, Shenzhen, China

**Keywords:** insomnia, pruritus, constipation, hemodialysis, quality of life

## Abstract

**Background:**

Insomnia, pruritus, and constipation are among the most prevalent chronic symptoms in hemodialysis patients, significantly impairing their quality of life. However, their risk factors and interrelationships are unclear. This study aimed to investigate the potential interrelationships among insomnia, pruritus, and constipation, as well as their associations with clinical and laboratory parameters in patients undergoing maintenance hemodialysis.

**Methods:**

Sleep quality was evaluated using the Athens Insomnia Scale, while pruritus was assessed based on patient-reported occurrences in the past 4 weeks. Constipation was diagnosed according to the Rome IV criteria for functional gastrointestinal disorders. Additional clinical and laboratory parameters were collected for comprehensive analysis. Statistical analyses included the t-test, Mann-Whitney U test, chi-square test, multivariate logistic regression,and receiver operating characteristic curve.

**Results:**

A total of 210 patients were included in this study. Insomnia was reported in 114 patients (54.3%), pruritus in 86 (41.0%), and constipation in 36 (17.1%). Patients with insomnia exhibited significantly higher serum calcium levels than those with normal sleep (P = 0.029). Insomnia was more prevalent among patients with pruritus (P = 0.009) and constipation (P = 0.006). Binary logistic regression identified elevated calcium levels (P = 0.045; OR = 3.613), pruritus (P = 0.014; OR = 2.078), and constipation (P = 0.012; OR = 2.882) as independent risk factors for insomnia. Hemodialysis patients with pruritus presented with elevated pre-dialysis creatinine (P = 0.002), post-dialysis creatinine (P = 0.012), post-dialysis urea levels (P = 0.035), creatinine clearance during dialysis (P = 0.001), ultrafiltration (P = 0.013), and ultrafiltration rate (P = 0.008) compared to those without pruritus. Insomnia (OR = 2.012, P = 0.019) and creatinine clearance during dialysis (OR = 1.002, P = 0.018) were identified as independent risk factors for patients with pruritus. Constipated patients exhibited significantly lower dialysis urea clearance than non-constipated patients (P = 0.005). Dry weight was higher in the constipated group (P = 0.024), and the prevalence of insomnia was significantly elevated compared to non-constipated patients (P = 0.006). Binary logistic regression analysis identified insomnia as an independent risk factor for constipation (P = 0.006; OR 3.253). Conversely, higher urea clearance during dialysis served as a protective factor against constipation (P = 0.013; OR 0.883). ROC curve analyses revealed AUC values of 0.59 for serum calcium in diagnosing insomnia, 0.64 for creatinine clearance during dialysis in diagnosing pruritus, and 0.65 for urea clearance during dialysis in diagnosing constipation.

**Conclusion:**

Insomnia, pruritus, and constipation demonstrate both complex interrelationships and independent effects, collectively contributing to substantial quality-of-life impairment in maintenance hemodialysis patients.

## 1 Introduction

With the improvement of hemodialysis technology in recent years, the mortality rate of hemodialysis patients has gradually decreased (McCullough et al., 2025). However, with increased dialysis duration, chronic complications that affect the quality of life of the patients become ignificant concerns for both nephrologists and patients.

In hemodialysis patients, chronic symptoms such as insomnia, pruritus and constipation are often overlooked compared to acute complications like electrolyte disturbances, heart failure and dialysis imbalance. Recent research demonstrates that insomnia, pruritus and constipation increase patient mortality by 16% (Elder et al., 2008), 23% (Kimata et al., 2014) and 14% (Park et al., 2025) respectively. These chronic symptoms are not only highly prevalent, but studies (Fletcher et al., 2022; Lowney et al., 2015) have confirmed their severe impact on patients’ quality of life. Although potential associations have been suggested with factors including hyperphosphatemia, low Kt/V, inflammation and malnutrition (Park et al., 2025; Ko et al., 2013; Chiu et al., 2008; Orasan et al., 2020), these relationships remain unconfirmed due to inconsistent statistical significance across studies (Weisshaar et al., 2015; Mucsi et al., 2004).

Current research lacks a comprehensive investigation into the risk factors for these chronic symptoms, particularly regarding their co-occurrence. This knowledge gap hinders nephrologists’ ability to develop effective therapeutic strategies, resulting in suboptimal treatment outcomes and persistent symptoms in patients (Scherer et al., 2017). Our study hypothesizes that insomnia, pruritus, and constipation are interrelated and co-occur more frequently than expected by chance, with inadequate dialysis clearance or calcium-phosphate metabolism disorders potentially serving as independent risk factors for these concurrent symptoms.

## 2 Subjects and methods

This is a single-center, cross-sectional study, which included maintenance hemodialysis (MHD) patients in Luohu Hospital from June 2024 to December 2024. Patient were treated by high flux hemodialysis or on-line hemodialysis filtration using Fresenius or Campbell dialysis machines. Inclusion criteria: age ≥ 18 years, maintenance hemodialysis ≥ 3 months (2–3 times per week for 3–4 h). Exclusion criteria: lack of clinical test data, acute comorbidities (infections, cardiovascular events, other acute illnesses), psychiatric disorders. This cross-sectional study employed the standard sample size calculation formula: n = Z^2^ × [P × (1 - P)]/E^2^, where: Z = 1.96 (for 95% confidence interval); P = expected prevalence (derived from literature); E = 0.1 (margin of error). Based on previous studies reporting prevalence rates of 49% for insomnia (Elder et al., 2008), 37% for pruritus (Sukul et al., 2021), and 53% for constipation (Murtagh et al., 2007) in hemodialysis populations, the minimum required sample sizes were calculated as 96, 90, and 96 patients, respectively. Our study enrolled 210 participants, providing adequate power to detect the hypothesized associations.

Sleep quality was evaluated using the Athens Insomnia Scale (AIS). The AIS was administered to participants immediately before their dialysis sessions. Study personnel were available to assist respondents in completing the questionnaire as needed. A score of <4 suggested no sleep disorder, and a score of ≥4 suggested insomnia. To comprehensively assess pruritus severity, patients rated their degree of itch-related distress by answering: “How much have you been bothered by itching in the past 4 weeks?” using a 5-point Likert scale: (1) not bothered at all, (2) somewhat bothered, (3) moderately bothered, (4) very bothered, and (5) extremely bothered. We assessed patients for constipation using a questionnaire based on the Rome IV diagnostic criteria for functional gastrointestinal disorders. Patients on chronic laxatives who met the Rome IV criteria before taking laxatives were categorized in the constipation group.

Data collected included patients’ gender, age, duration of dialysis, type of vascular access, presence of diabetes, presence of hypertension, presence of cardiac disease,dry weight, dialysis time per week, ultrafiltration (UF) volume, UF rate, SpKt/V (single-pool Kt/V), hemoglobin (Hb), pre-dialysis creatinine (Cr),urea, uric acid, albumin,intact parathyroid hormone (iPTH), high-sensitivity C-reactive protein (hs-CRP), calcium (Ca), phosphate(P),sodium (Na),chloride (Cl),magnesium (Mg),beta 2-microglobulin (β2-MG), ferritin, carbon dioxide combining power (CO2CP),white blood cell count (WBC),platelet count (PLT),serum prealbumin, post-dialysis creatinine, post-dialysis urea, creatinine clearance during dialysis, and urea clearance during dialysis, along with other clinical and biochemical parameters. All blood samples were collected prior to dialysis initiation on the treatment day, with the exception of post-dialysis creatinine and urea levels, which were obtained following dialysis completion. The post-dialysis sampling protocol was performed as follows: First, the ultrafiltration rate was set to zero, and the dialysate was switched to bypass mode while maintaining blood flow at the standard rate for 3–5 min. Subsequently, blood specimens were drawn from the arterial or venous line.

## 3 Statistics

All statistical analyses were performed using SPSS software (version19.0). Continuous variables with normal distribution were compared using independent Student's t-tests, while the Mann-Whitney U test was employed for non-normally distributed data. Categorical variables were analyzed using Pearson’s chi-square tests. After excluding covariates, variables with statistically significant (P < 0.05) results from the univariate analyses described above were entered into a binary logistic regression model to identify independent risk factors and assess their association with clinical outcomes. The discriminative performance of the significant predictors was evaluated using receiver operating characteristic curve (ROC) analysis. A two-tailed P-value <0.05 was considered statistically significant for all analyses.

## 4 Results

**FIGURE 1:**
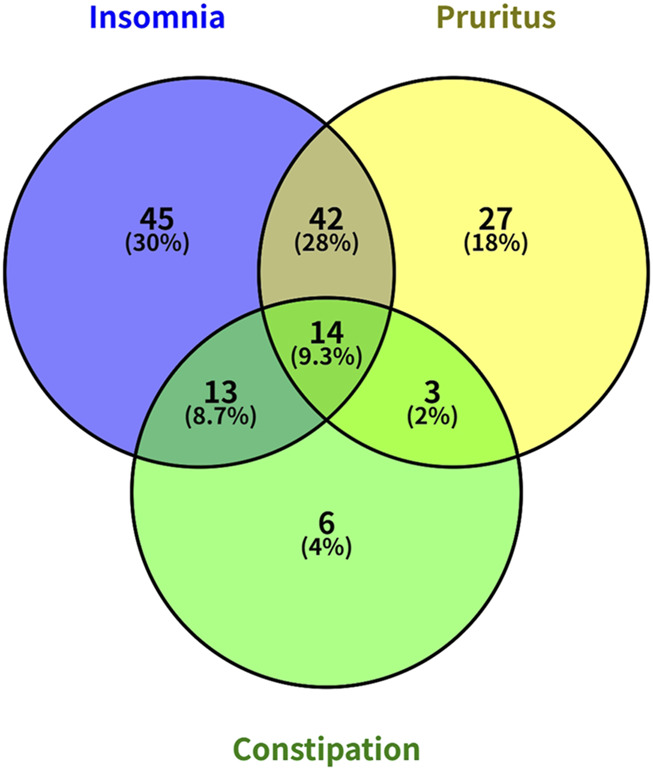
Venn diagram illustrating the co-occurrence of insomnia, pruritus, and constipation among MHD patients.

A total of 210 MHD patients were included in this study, consisting of 138 males (65.71%) and 72 females (34.29%), with an average age of 56.29 ± 13.27 years and an average duration of dialysis of 59.05 ± 47.29 months. Baseline characteristics of primary renal diseases and complications in MHD patients were listed in Table 1. Among these patients, 181 (86.20%) used arteriovenous fistulas (AVF), while 29 (13.80%) required catheter dialysis. 82 patients (39.05%) had diabetes. Insomnia was reported in 114 patients (54.29%), pruritus in 86 patients (40.95%), and constipation in 36 patients (17.14%). 60 (28.57%) patients were free from insomnia, pruritus, and constipation; 78 (37.14%) patients were affected by one of these conditions; 58 (27.62%) patients were affected by two conditions; and 14 (6.67%) were affected by all three Figure 1.

**TABLE 1:** Baseline characteristics of primary renal diseases and complications in MHD patients.

Category	Number (n)	Percentage (%)
Total patients	210	100.00%
Primary disease		
Chronic glomerulonephritis	73	34.76%
Diabetic nephropathy	82	39.05%
Obstructive nephropathy	8	3.81%
Polycystic kidney disease	7	3.33%
Others	40	19.05%
Complications		
Hypertension	184	87.62%
Cardiac disease	57	27.14%
Cerebrovascular disease	30	14.29%
Hypercalcemia	21	10.00%
Hypocalcemia	56	26.67%
Hyperphosphatemia	142	67.62%
Hyperkalemia	22	10.48%

In our study, 54.29% of patients reported experiencing insomnia and 26.67% of patients requiring hypnotic medications.47.1% had difficulty falling asleep, 46.19% had awakening from sleep, 44.76% had early awakening and 37.62% had all of the above. Patients with insomnia had higher blood calcium than patients with normal sleep (P = 0.029). Patients with pruritus (P = 0.009) and constipation (P = 0.006) had higher rates to suffer from insomnia (Table 2). Calcium (OR = 3.613, 95% CI: 1.0–12.67, P = 0.045), pruritus (OR = 2.078, 95% CI: 1.16–3.72, P = 0.014), and constipation (OR = 2.88, 95% CI: 1.26–6.60, P = 0.012) had been identified as independent risk factors for insomnia, with results remaining significant after adjusting for confounders (Table 3). No significant differences were observed in other clinical and laboratory indicators.

**TABLE 2:** Demographic and clinical characteristics of maintenance hemodialysis patients.

Baseline characteristics	All patients N = 210	Insomnia		P	Pruritus		P	Constipation		P
		Yes N = 114	No N = 96		Yes N = 86	No N = 124		Yes N = 36	No N = 174	
Men, n (%)	138 (65.71%)	76 (66.67%)	62 (64.58%)	0.751	62 (72.09%)	76 (61.30%)	0.105	25 (69.44%)	113 (64.94%)	0.604
Diabetes, n (%)	82 (39.05%)	45 (39.48%)	37 (38.54%)	0.890	29 (33.72%)	53 (42.74%)	0.188	17 (47.22%)	65 (37.35%)	0.269
Hypertension, n (%)	184 (87.62%)	100 (8.72)	84 (87.50%)	0.962	74 (86.04%)	110 (88.71%)	0.564	36 (91.67%)	151 (86.78%)	0.418
Cardiac diseasen, n (%)	57 (27.14%)	32 (28.07%)	25 (26.04%)	0.742	18 (20.93%)	39 (31.45%)	0.092	10 (27.78%)	47 (27.01%)	0.925
AVF, n (%)	181 (86.20%)	101 (88.60%)	80 (83.33%)	0.271	76 (88.37%)	105 (84.68%)	0.445	30 (83.33%)	151 (86.78%)	0.585
Age (year)	56.29 ± 13.27	56.26 ± 11.30	56.31 ± 15.27	0.979	55.79 ± 12.41	56.63 ± 13.88	0.654	57.14 ± 13.31	56.11 ± 13.30	0.673
Duration of MHD (months)	59.05 ± 47.29	56.87 ± 44.70	61.65 ± 50.30	0.726	58.3 ± 48.78	59.57 ± 46.42	0.714	54.03 ± 44.67	60.09 ± 47.87	0.513
Dialysis time per week (hours)	11.27 ± 1.37	11.18 ± 1.42	11.38 ± 1.30	0.269	11.16 ± 1.39	11.35 ± 1.36	0.532	11.47 ± 1.10	11.23 ± 1.42	0.569
Dry weight (kg)	62.4 ± 13.41	62.19 ± 12.55	62.65 ± 14.42	0.958	62.43 ± 12.28	62.38 ± 14.18	0.771	66.04 ± 13.11	61.65 ± 13.38	0.024
UF volume (ml)	2728.11 ± 831.54	2763.10 ± 850.24	2668.56 ± 811.24	0.508	2899.03 ± 748.99	2609.57 ± 867.55	0.013	2816.76 ± 779.07	2709.77 ± 842.96	0.484
UF rate (mL/min)	11.71 ± 3.58	11.91 ± 3.66	11.47 ± 3.47	0.368	12.50 ± 3.25	11.17 ± 3.70	0.008	12.20 ± 3.36	11.61 ± 3.62	0.372
SpKt/V	1.31 ± 0.25	1.30 ± 0.23	1.31 ± 0.27	0.669	1.283 ± 0.24	1.323 ± 0.26	0.235	1.24 ± 0.22	1.32 ± 0.26	0.100
Hb (g/L)	109.75 ± 16.75	109.19 ± 17.22	110.42 ± 16.23	0.599	107.873 ± 18.72	111.06 ± 15.18	0.176	39.68 ± 2.94	39.44 ± 3.16	0.983
K (mmol/L)	39.48 ± 3.11	4.76 ± 0.71	4.73 ± 0.63	0.793	4.83 ± 0.66	4.693 ± 0.68	0.198	4.73 ± 0.60	4.75 ± 0.69	0.953
Ca (mmol/L)	2.21 ± 0.23	2.24 ± 0.26	2.17 ± 0.18	0.029	2.22 ± 0.25	2.20 ± 0.23	0.423	2.23 ± 0.24	2.20 ± 0.23	0.724
P (mmol/L)	2.00 ± 0.53	2.04 ± 0.55	1.9 ± 0.50	0.210	2.06 ± 0.47	1.96 ± 0.56	0.140	1.97 ± 0.41	2.01 ± 0.55	0.698
Na (mmol/L)	136.50 ± 3.39	136.50 ± 3.39	136.36 ± 3.48	0.428	136.79 ± 3.40	136.31 ± 3.39	0.277	136.94 ± 3.12	136.41 ± 3.45	0.62
Cl (mmol/L)	95.94 ± 4.00	95.94 ± 4.00	95.79 ± 4.29	0.546	96.08 ± 4.14	95.85 ± 3.92	0.677	96.47 ± 4.00	95.83 ± 4.01	0.385
Mg (mmol/L)	1.09 ± 0.14	1.10 ± 0.15	1.08 ± 0.14	0.363	1.11 ± 0.13	1.08 ± 0.15	0.144	1.07 ± 0.16	1.10 ± 0.14	0.292
CO2CP(mmol/L)	19.52 ± 2.60	19.42 ± 2.50	19.65 ± 2.72	0.511	19.49 ± 2.43	19.55 ± 2.73	0.864	19.68 ± 2.34	19.49 ± 2.66	0.704
Uric acid (μmol/L)	443.86 ± 96.04	450.20 ± 101.11	436.92 ± 89.60	0.426	458.11 ± 99.54	433.97 ± 92.66	0.052	430.22 ± 93.33	446.68 ± 96.62	0.48
iPTH (pmol/L)	49.69 ± 49.00	44.96 ± 38.44	55.31 ± 58.87	0.187	46.69 ± 40.18	51.77 ± 54.35	0.452	62.82 ± 64.93	46.98 ± 44.77	0.271
hs-CRP (mg/L)	4.38 ± 6.36	4.70 ± 7.21	4.00 ± 5.18	0.983	4.33 ± 5.72	4.41 ± 6.79	0.534	3.71 ± 5.45	4.52 ± 6.54	0.213
Albumin (g/L)	39.48 ± 3.11	39.67 ± 2.85	39.25 ± 3.40	0.457	40.06 ± 2.87	39.08 ± 3.23	0.067	39.68 ± 2.94	39.44 ± 3.16	0.870
Prealbumin (mg/L)	352.62 ± 82.54	352.62 ± 82.54	359.78 ± 87.70	0.172	360.53 ± 81.83	347.14 ± 82.92	0.248	39.68 ± 2.94	39.44 ± 3.16	0.607
β2-MG (mg/L)	29.24 ± 6.66	30.06 ± 6.91	28.26 ± 6.244	0.055	30.04 ± 6.00	28.69 ± 7.05	0.186	28.03 ± 6.83	29.49 ± 6.62	0.064
Ferritin (ng/mL)	319.14 ± 569.08	306.55 ± 428.49	334.08 ± 702.62	0.903	431.96 ± 814.66	240.89 ± 276.74	0.551	218.05 ± 258.01	340.05 ± 612.54	0.192
WBC(×109/L)	6.47 ± 1.89	6.40 ± 1.75	6.54 ± 2.05	0.726	6.29 ± 1.91	6.59 ± 1.88	0.145	6.72 ± 2.19	6.41 ± 1.83	0.534
PLT (×109/L)	196.49 ± 65.66	195.59 ± 71.04	197.55 ± 58.99	0.615	191.19 ± 68.43	200.16 ± 63.69	0.191	189.97 ± 64.57	197.83 ± 65.99	0.582
Pre-dialysis Cr (μmol/L)	997.39 ± 239.39	1019.08 ± 247.53	971.62 ± 227.94	0.161	1063.13 ± 199.02	951.79 ± 254.73	0.002	990.72 ± 225.57	998.76 ± 242.75	0.819
Pre-dialysis Urea (mmol/L)	25.12 ± 6.50	25.13 ± 6.52	25.11 ± 6.52	0.965	25.88 ± 6.15	24.6 ± 6.71	0.091	23.33 ± 5.31	25.49 ± 6.68	0.055
Post-dialysis Cr (μmol/L)	392.99 ± 126.36	402.55 ± 131.45	381.63 ± 119.72	0.261	418.37 ± 117.95	375.39 ± 129.43	0.012	410.39 ± 116.85	389.39 ± 128.26	0.292
Post-dialysis Urea (mmol/L)	8.78 ± 3.37	8.69 ± 3.41	8.86 ± 3.34	0.561	9.21 ± 3.21	8.48 ± 3.45	0.035	8.60 ± 2.66	8.82 ± 3.50	0.906
Cr clearance (μmol/L)	602.76 ± 157.16	613.52 ± 160.63	589.99 ± 152.79	0.147	640.65 ± 142.92	576.48 ± 161.72	0.001	570.53 ± 162.72	609.43 ± 155.63	0.176
Urea clearance (mmol/L)	16.34 ± 4.15	16.27 ± 4.07	16.42 ± 4.25	0.909	16.67 ± 3.97	16.11 ± 4.27	0.321	14.72 ± 3.61	16.68 ± 4.19	0.005
Insomnia	112 (53.33%)	—	—	—	56 (65.11%)	58 (46.78%)	0.009	27 (75.00%)	9 (50.00%)	0.006
Pruritus	86 (40.95%)	56 (49.12%)	30 (31.25%)	0.009	—	—	—	17 (47.22)	69 (39.66)	0.401
Constipation	36 (17.14%)	27 (23.68%)	9 (9.38%)	0.006	17 (19.77%)	19 (15.32%)	0.401	—	—	—

BMI, body mass index; AVF, arteriovenous fistulas; MHD, maintenance hemodialysis patient; UF, ultrafiltration; SpKt/V, single-pool hemodialysis adequacy; Hb, hemoglobin; Ca, calcium; P, phosphate; Na, sodium; Cl, chloride; Mg, Magnesium; CO2CP, carbon dioxide combining pow; iPTH, intact parathyroid hormone; hs-CRP, high-sensitivity C-reactive protein; β2-MG, beta 2-microglobulin; WBC, white blood cell count; PLT, platelet count; Cr, creatinine.

**TABLE 3:** Binary logistic regression analysis of risk factors for insomnia.

Predictor variables	B	SE	OR (95% CI)	P	VIF
Calcium (mmol/L)	1.285	0.640	3.613 (1.031–12.661)	0.045	1.003
Pruritus	0.732	0.297	2.078 (1.161–3.721)	0.014	1.005
Constipation	1.059	0.423	2.882 (1.26–6.601)	0.012	1.004

Hosmer-Lemeshow Test: χ^2^ = 5.77, p = 0.673.

40.95% of MHD patients experienced pruritus and 37.2% requiring antihistamine medications. Pruritus hemodialysis patients had higher pre-dialysis creatinine (P = 0.002), post-dialysis creatinine (P = 0.012), post-dialysis urea (P = 0.035), creatinine clearance during dialysis (P = 0.001), ultrafiltration (P = 0.013) and ultrafiltration rate (P = 0.008) than patients without pruritus (Table 2). Binary logistic regression analysis identified two independent risk factors in hemodialysis patients: insomnia (OR = 2.01, 95% CI: 1.12–3.61, p = 0.019) and dialytic creatinine clearance (OR = 1.002, 95% CI: 1.000–1.004, p = 0.018) (Table 4). 17.14% MHD patients had constipation and 10.00% MHD patients required laxative medication. Urea clearance during dialysis was lower in constipated patients than in patients without constipation (P = 0.005). Dry weight was higher in constipated patients (P = 0.024) and insomnia was more in constipated patients (P = 0.006) (Table 2). Insomnia was an independent risk factor for constipation (OR = 3.253, 95% CI: 1.41–7.50, P = 0.006). High urea clearance during dialysis was a protective factor for constipation (OR = 0.883, 95% CI: 0.80–0.974, P = 0.013) (Table 5).

**TABLE 4:** Binary logistic regression analysis of risk factors for pruritus.

Predictor variables	B	SE	OR (95% CI)	P	VIF
UF rate (mL/min)	0.082	0.047	1.086 (0.991–1.190)	0.077	1.256
Post-dialysis Urea (mmol/L)	0.021	0.048	1.021 (0.930–1.121)	0.663	1.226
Cr clearance (μmol/L)	0.002	0.001	1.002 (1.000–1.004)	0.018	1.037
insomnia	0.699	0.298	2.012 (1.122–3.607)	0.019	1.008

Hosmer-Lemeshow Test: χ^2^ = 7.651, p = 0.468.

UF, ultrafiltration; Cr,creatinine.

**TABLE 5:** Binary logistic regression analysis of risk factors for constipation.

Predictor variables	B	SE	OR (95% CI)	P	VIF
Dry weight (kg)	0.003	0.002	1.025 (0.998–1.055)	0.071	1.016
Urea clearance (mmol/L)	−0.014	0.006	0.883 (0.800–0.974)	0.013	1.012
insomnia	0.087	0.026	3.253 (1.410–7.503)	0.006	1.005

Hosmer-Lemeshow Test: χ^2^ = 9.13, p = 0.331.

**FIGURE 2:**
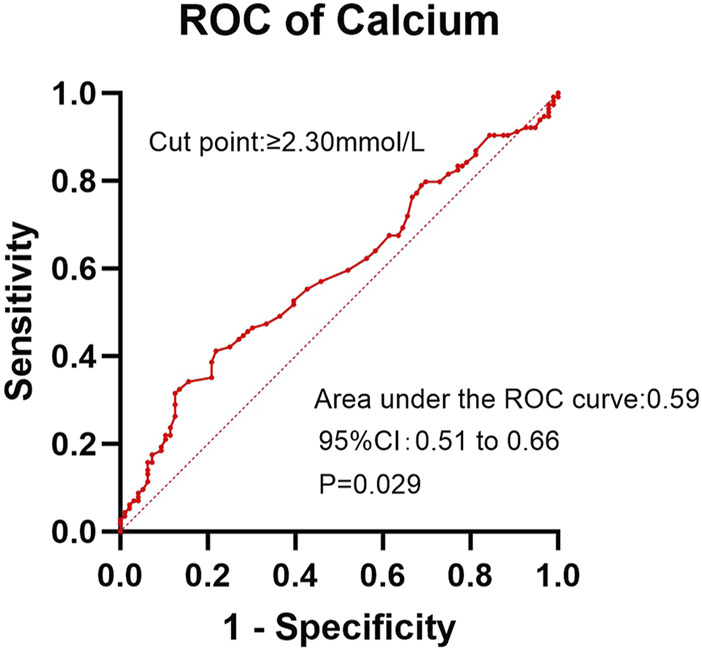
Diagnostic performance of calcium for insomnia.

**FIGURE 3:**
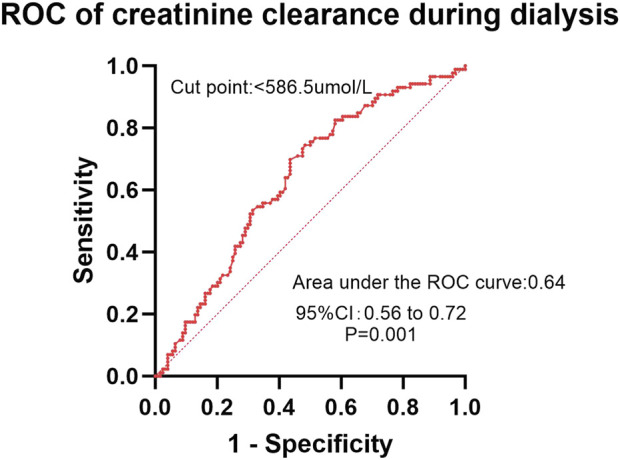
Diagnostic performance of creatinine clearance during dialysis for pruritus.

**FIGURE 4:**
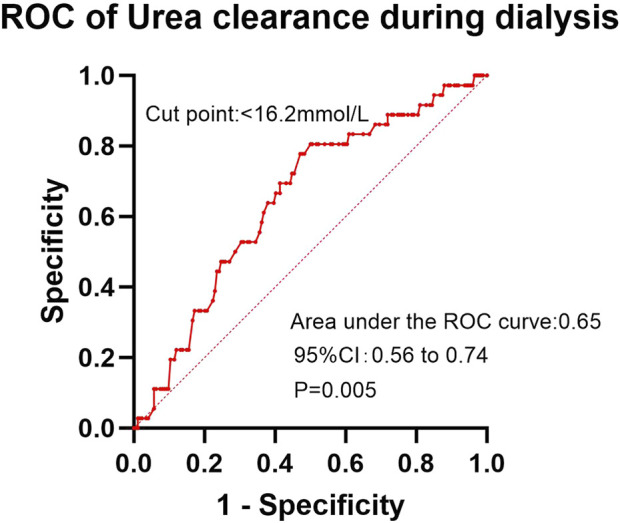
Diagnostic performance of urea clearance during dialysis for constipation.

In the ROC analysis evaluating the diagnostic performance of serum calcium levels for insomnia, the area under the curve (AUC) was 0.59 (95% CI: 0.51–0.66; P = 0.029). The optimal cutoff point was >2.30 mmol/L, yielding a sensitivity of 41.2% (95% CI: 32.2%–50.8%) and a specificity of 78.1% (95% CI: 68.4%–85.7%) (Figure 2). For creatinine clearance during dialysis as a predictor of pruritus, the ROC analysis revealed an AUC of 0.64 (95% CI: 0.56–0.72; P = 0.001). A cutoff value of <586.5 μmol/L provided a sensitivity of 69.8% (95% CI: 59.6%–78.3%) and a specificity of 56.5% (95% CI: 47.8%–64.9%) (Figure 3). Similarly, urea clearance during dialysis for constipation showed an AUC of 0.65 (95% CI: 0.56–0.74; P = 0.005). The identified threshold was <16.2 mmol/L, with a sensitivity of 77.8% (95% CI: 61.1%–89.0%) and a specificity of 52.9% (95% CI: 45.3%–60.4%) (Figure 4).

## 5 Discussion

Insomnia, pruritus and constipation are common chronic symptoms among MHD patients, which severely reduce the quality of life. However, researchers often study laboratory markers or all-cause deaths, and there is a lack of attention to symptoms that affect the quality of life of dialysis patients. The causes of these symptoms are still unclear, and most physicians can only provide medication treatments to relieve the symptoms. It is important to clarify risk factors, laboratory indicators and understand the relationship between them.

There has been a high prevalence of insomnia in hemodialysis patients. Early in 2008, Dialysis Outcomes and Practice Patterns Study (DOPPS) reported that 49% of hemodialysis patients had sleep disorders (Elder et al., 2008). Insomnia may lead to depression, immune dysfunctions and cardiovascular events (Cukor et al., 2021). The underlying cause of insomnia in dialysis patients remains unclear. However, dialysis-related factors, such as fragmented sleep during dialysis sessions and prolonged periods away from home, may contribute to sleep disturbances (Morin et al., 2015). The mechanism of insomnia in MHD people may be related to high levels of toxins (subclinical uremic encephalopathy) and circadian dysregulation of melatonin secretion. Studies have reported the associations between insomnia in uremic patients and conditions such as diabetes, coronary heart diseases, heart failure, peripheral artery diseases, high body mass index (BMI) and depression (Koch et al., 2008). Evidence suggests that both behavioral therapies and pharmacologic interventions for sleep improvement demonstrate limited efficacy, with effects comparable to placebo in this population (Mehrotra et al., 2024). In our study, the prevalence of insomnia among dialysis patients was 54.29%, consistent with rates reported in prior research. However, unlike the DOPPS study, which identified comorbid heart disease, diabetes mellitus, and hyperphosphatemia as significant risk factors for insomnia, our analysis did not demonstrate these associations. These discrepancies may stem from differences in population demographics, geographic variations, or comorbidities. Additionally, as a single-center study, our findings require further validation through large-scale, multicenter investigations. Our study identified elevated serum calcium levels as a significant independent risk factor for insomnia in hemodialysis patients. The relationship between calcium and sleep remains poorly characterized, particularly in hemodialysis populations. The observed association may reflect underlying physiological mechanisms, as experimental studies have demonstrated that calcium-dependent hyperpolarization pathways play an important role in regulating sleep duration in mammalian models (Tatsuki et al., 2016). Astrocytes, the most abundant glial cells in the central nervous system, exhibit dynamic calcium signaling that varies with sleep-wake states. Specifically, astrocytic calcium activity is highest during wakefulness and lowest during sleep, indicating a critical role for intracellular Ca^2+^ signaling in sleep regulation (Bojarskaite et al., 2020). To investigate diurnal fluctuations in systemic calcium levels, one study measured total serum calcium in healthy volunteers during nighttime and daytime sleep. Blood samples were collected hourly for calcium analysis. The results revealed a median intra-individual coefficient of variation of 3.3% during nighttime sleep compared to 2.8% during daytime sleep. Notably, serum calcium displayed significant diurnal variation, with the lowest levels (trough) occurring at the end of the nighttime sleep period. The amplitude of this fluctuation was approximately 0.07 mmol/L between the trough and peak. Importantly, calcium levels were consistently lower during both nighttime and daytime sleep periods than during wakefulness (Ridefelt et al., 2012). This physiological pattern suggests calcium homeostasis may play an important modulatory role in sleep regulation. High calcium may lead to abnormal regulation of insomnia, which requires more research. Pruritus was identified as an independent risk factor for insomnia. This association may be mediated through pruritus-induced nocturnal discomfort, which can significantly impair sleep initiation and maintenance. The resulting sleep fragmentation likely contributes to the development of chronic insomnia in affected patients. Constipation exacerbates insomnia because brain and gastrointestinal function are closely linked, including digestive regulation and the gut immune system (Carabotti et al., 2015). The gut-brain axis constitutes a bidirectional communication system between the central and enteric nervous systems, mediated through metabolic, immune, and neuronal pathways (Wang Z. et al., 2022). Insomnia induces both dysfunction and compositional alterations in the gut microbiota. Specifically, insomnia patients demonstrate significantly increased proportions of *Lactobacillus*, *Streptococcus*, and *Bacteroides fragilis* compared to healthy controls (Wang Q. et al., 2022). To investigate microbial involvement, researchers administered broad-spectrum antibiotics to mice, resulting in altered sleep/wake architecture and EEG power spectra after 4 weeks (Ogawa et al., 2020). Constipation-positive maintenance hemodialysis patients exhibit distinct gut microbiota profiles compared to their non-constipated counterparts (Zhang et al., 2024). Moreover, intestinal symptoms can reciprocally impair sleep quality, as evidenced by the significantly poorer sleep observed in patients with gastroesophageal reflux disease and irritable bowel syndrome (Morito et al., 2014).

DOPPS suggests that 37% of hemodialysis patients have pruritus (Sukul et al., 2021). Severe pruritus has persisted for years in patients and is recognized as an independent risk factor for mortality. The exact causes of pruritus remain unclear. Studies suggest that it may be associated with factors such as inflammation, inadequate dialysis, catheter use, chronic kidney disease-mineral and bone disorder (CKD-MBD), malnutrition, anemia, older age, and male gender (Ko et al., 2013; Chiu et al., 2008; Pisoni et al., 2006). Additionally, our research found that pruritus was associated not only with pre-dialysis creatinine, post-dialysis creatinine,post-dialysis urea, creatinine clearance in dialysis but also with ultrafiltration. Patients experiencing pruritus had higher ultrafiltration volume and ultrafiltration rate compared to those without pruritus. Previous studies on the relationship between pruritus and the ultrafiltration are scarce, with only the study by Ozer H et al. having similar results (Ozer et al., 2024). Pruritus has been thought to be associated with high phosphorus, high iPTH, and low SpKt/V levels (Rayner et al., 2017). However, some studies, including ours, have indicated no correlation. Pruritus was strongly associated with sleep, and pruritus in MHD patients was associated with a 17% increased risk of death, which was no longer significant after adjustment for sleep (Pisoni et al., 2006).

The prevalence of constipation in the general population is about 16% (Mugie et al., 2011), while among hemodialysis patients, it ranges from 25.9% to 53%, significantly higher than in the general population (Murtagh et al., 2007). Constipation elevates cardiovascular risk in the general population and is associated with a 14% increase in all-cause mortality among hemodialysis patients (Park et al., 2025; Sundbøll et al., 2020). Long-term use of laxatives also increases the risk of cardiovascular events and death (Honda et al., 2021). There are other adverse effects of constipation, including depression, anxiety, poor appetite, and diminished intestinal detoxification. The causes of constipation in hemodialysis patients may include restrictions on fiber and fluid intake, the use of medications (such as potassium binders and phosphate binders), and dysbiosis of the gut microbiome (Yasuda et al., 2002). Study has shown that constipation increases uremic toxins (Ramos et al., 2020). In turn, toxin removal may improve bowel function. In our study high clearance of urea in dialysis was a protective factor for constipation, suggesting that increased clearance of toxins such as urea improves constipation. We also found that patients with higher dry weights experienced more constipation, possibly due to stricter dietary and fluid management. Insomnia is a risk factor for constipation (Yun et al., 2022), potentially mediated through autonomic nervous system dysregulation (Tian et al., 2024).

Although the AUC values in the ROC curve analysis (ranging from 0.59 to 0.65) indicate limited discriminatory power, these findings remain clinically relevant. Previous studies have failed to establish definitive conclusions regarding the factors influencing insomnia, pruritus, and constipation in this population. Even with modest AUC values, our results provide moderate clinical utility, as they suggest that the model performs better than chance alone. However, large-scale multicenter studies with expanded sample sizes are warranted to validate these observations and explore the underlying mechanisms more comprehensively.

Based on our findings, a multimodal therapeutic approach targeting calcium homeostasis and improved clearance of uremic toxins may be effective in relieving concurrent symptoms of insomnia, pruritus, and constipation in dialysis patients. This strategy should include (1) downregulation of blood calcium by low-calcium dialysate (1.25–1.5 mmol/L) and reduction of calcium-based phosphate binders, and (2) increased uremic toxin clearance by prolonging the duration of treatment and increasing the surface area of the dialyzer. Importantly, our observations suggest that these interventions may have a synergistic effect, as improvement in one symptom may improve other symptom areas and ultimately improve quality of life.

Several limitations of this study warrant consideration. First, the single-center, cross-sectional design with a moderate sample size (n = 210) may affect the external validity of our findings. Second, the assessment relied on patient-reported outcomes for insomnia, pruritus, and constipation - symptoms that demonstrate considerable inter-individual variability in both perception and tolerance thresholds. Most importantly, the observational nature of this study precludes establishment of causal relationships among these frequently comorbid symptoms. Future research should employ longitudinal designs and interventional approaches to better characterize the pathophysiology of uremia-associated chronic symptoms and their impact on long-term clinical outcomes.

## 6 Conclusion

Insomnia, pruritus, and constipation demonstrate both complex interrelationships and independent effects, collectively contributing to substantial quality-of-life impairment in MHD patients. These clinically significant chronic conditions warrant comprehensive investigation to elucidate both their distinct and shared pathophysiological mechanisms.

## Data Availability

The raw data supporting the conclusions of this article will be made available by the authors, without undue reservation.

## Abbreviations

MHD, maintenance hemodialysis patient; AIS, Athens Insomnia Scale; UF, Ultrafiltration; SpKt/V, single-pool Kt/V; Hb, hemoglobin; Cr, creatinine; iPTH, intact parathyroid hormone; hs-CRP, high-sensitivity C-reactive protein; Ca, Calcium; P, phosphate; Na, sodium; Cl, chloride; Mg, Magnesium; β2-MG, beta 2-microglobulin; CO2CP, carbon dioxide combining power; WBC, white blood cell count; PLT, platelet count; ROC, receiver operating characteristic curve; AVF, arteriovenous fistulas; AUC, area under the curve; OR, odds ratio; 95%CI, 95% confidence interval; DOPPS, Dialysis Outcomes and Practice Patterns Study; BMI, body mass index.

## References

[B1] Bojarskaite L. Bjørnstad D. M. Pettersen K. H. Cunen C. Hermansen G. H. Åbjørsbråten K. S. (2020). Astrocytic Ca(2+) signaling is reduced during sleep and is involved in the regulation of slow wave sleep. Nat. Commun. 11 (1), 3240. 10.1038/s41467-020-17062-2 32632168 PMC7338360

[B2] Carabotti M. Scirocco A. Maselli M. A. Severi C. (2015). The gut-brain axis: interactions between enteric microbiota, central and enteric nervous systems. Ann. gastroenterology 28 (2), 203–209. Available online at: https://pmc.ncbi.nlm.nih.gov/articles/PMC4367209 . PMC4367209 25830558

[B3] Chiu Y. L. Chen H. Y. Chuang Y. F. Hsu S. P. Lai C. F. Pai M. F. (2008). Association of uraemic pruritus with inflammation and hepatitis infection in haemodialysis patients. Nephrol. Dial. Transplant. 23 (11), 3685–3689. 10.1093/ndt/gfn303 18515654

[B4] Cukor D. Unruh M. McCurry S. M. Mehrotra R. (2021). The challenge of insomnia for patients on haemodialysis. Nat. Rev. Nephrol. 17 (3), 147–148. 10.1038/s41581-021-00396-5 33479446 PMC7818049

[B5] Elder S. J. Pisoni R. L. Akizawa T. Fissell R. Andreucci V. E. Fukuhara S. (2008). Sleep quality predicts quality of life and mortality risk in haemodialysis patients: results from the dialysis outcomes and practice patterns study (DOPPS). Nephrol. Dial. Transplant. 23 (3), 998–1004. 10.1093/ndt/gfm630 17911092

[B6] Fletcher B. R. Damery S. Aiyegbusi O. L. Anderson N. Calvert M. Cockwell P. (2022). Symptom burden and health-related quality of life in chronic kidney disease: a global systematic review and meta-analysis. PLoS Med. 19 (4), e1003954. 10.1371/journal.pmed.1003954 35385471 PMC8985967

[B7] Honda Y. Itano S. Kugimiya A. Kubo E. Yamada Y. Kimachi M. (2021). Laxative use and mortality in patients on haemodialysis: a prospective cohort study. BMC Nephrol. 22 (1), 363. 10.1186/s12882-021-02572-y 34732171 PMC8565050

[B8] Kimata N. Fuller D. S. Saito A. Akizawa T. Fukuhara S. Pisoni R. L. (2014). Pruritus in hemodialysis patients: results from the Japanese dialysis outcomes and practice patterns study (JDOPPS). Hemodial. Int. Int. Symposium Home Hemodial. 18 (3), 657–667. 10.1111/hdi.12158 24766224

[B9] Ko M. J. Wu H. Y. Chen H. Y. Chiu Y. L. Hsu S. P. Pai M. F. (2013). Uremic pruritus, dialysis adequacy, and metabolic profiles in hemodialysis patients: a prospective 5-year cohort study. PloS one 8 (8), e71404. 10.1371/journal.pone.0071404 23940749 PMC3735516

[B10] Koch B. C. Nagtegaal J. E. Hagen E. C. van Dorp W. T. Boringa J. B. Kerkhof G. A. (2008). Subjective sleep efficiency of hemodialysis patients. Clin. Nephrol. 70 (5), 411–416. 10.5414/cnp70411 19000541

[B11] Lowney A. C. Myles H. T. Bristowe K. Lowney E. L. Shepherd K. Murphy M. (2015). Understanding what influences the health-related quality of life of hemodialysis patients: a collaborative study in England and Ireland. J. pain symptom Manag. 50 (6), 778–785. 10.1016/j.jpainsymman.2015.07.010 26300026

[B12] McCullough K. P. Morgenstern H. Rayner H. C. Port F. K. Jadoul M. Y. Akizawa T. (2025). Explaining international trends in mortality on hemodialysis through changes in hemodialysis practices in the dialysis outcomes and practice patterns study (DOPPS). Am. J. kidney Dis. 85 (1), 25–35.e1. 10.1053/j.ajkd.2024.06.017 39127399

[B13] Mehrotra R. Cukor D. McCurry S. M. Rue T. Roumelioti M. E. Heagerty P. J. (2024). Effectiveness of existing insomnia therapies for patients undergoing hemodialysis: a randomized clinical trial. Ann. Intern. Med. 177 (2), 177–188. 10.7326/M23-1794 38224591

[B14] Morin C. M. Drake C. L. Harvey A. G. Krystal A. D. Manber R. Riemann D. (2015). Insomnia disorder. Nat. Rev. Dis. Prim. 1, 15026. 10.1038/nrdp.2015.26 27189779

[B15] Morito Y. Aimi M. Ishimura N. Shimura S. Mikami H. Okimoto E. (2014). Association between sleep disturbances and abdominal symptoms. Intern. Med. Tokyo, Jpn. 53 (19), 2179–2183. 10.2169/internalmedicine.53.2591 25274228

[B16] Mucsi I. Molnar M. Z. Rethelyi J. Vamos E. Csepanyi G. Tompa G. (2004). Sleep disorders and illness intrusiveness in patients on chronic dialysis. Nephrol. Dial. Transplant. 19 (7), 1815–1822. 10.1093/ndt/gfh130 15161955

[B17] Mugie S. M. Benninga M. A. Di Lorenzo C. (2011). Epidemiology of constipation in children and adults: a systematic review. Best Pract. and Res. Clin. gastroenterology 25 (1), 3–18. 10.1016/j.bpg.2010.12.010 21382575

[B18] Murtagh F. E. Addington-Hall J. Higginson I. J. (2007). The prevalence of symptoms in end-stage renal disease: a systematic review. Adv. chronic kidney Dis. 14 (1), 82–99. 10.1053/j.ackd.2006.10.001 17200048

[B19] Ogawa Y. Miyoshi C. Obana N. Yajima K. Hotta-Hirashima N. Ikkyu A. (2020). Gut microbiota depletion by chronic antibiotic treatment alters the sleep/wake architecture and sleep EEG power spectra in mice. Sci. Rep. 10 (1), 19554. 10.1038/s41598-020-76562-9 33177599 PMC7659342

[B20] Orasan O. H. Muresan F. Mot A. Sitar Taut A. Minciuna I. Coste S. C. (2020). Hemodialysis patients with pruritus and insomnia have increased risk of death. Blood Purif. 49 (4), 419–425. 10.1159/000505147 31910406

[B21] Ozer H. Ozturk Y. Yonet F. Baloglu I. Turkmen K. Selcuk N. Y. (2024). Overlooked factor in the etiology of pruritus in hemodialysis patients: ultrafiltration volume. Ther. Apher. dialysis 28 (2), 234–239. 10.1111/1744-9987.14087 37992762

[B22] Park S. C. Jung J. Kwon Y. E. Baeg S. I. Oh D. J. Kim D. H. (2025). Constipation and risk of death and cardiovascular events in patients on hemodialysis. Kidney Res. Clin. Pract. 44 (1), 155–163. 10.23876/j.krcp.24.174 39815794 PMC11838856

[B23] Pisoni R. L. Wikström B. Elder S. J. Akizawa T. Asano Y. Keen M. L. (2006). Pruritus in haemodialysis patients: international results from the dialysis outcomes and practice patterns study (DOPPS). Nephrol. Dial. Transplant. 21 (12), 3495–3505. 10.1093/ndt/gfl461 16968725

[B24] Ramos C. I. Armani R. G. Canziani M. E. Ribeiro Dolenga C. J. Nakao L. S. Campbell K. L. (2020). Bowel habits and the association with uremic toxins in non-dialysis-dependent chronic kidney disease patients. J. Ren. Nutr. 30 (1), 31–35. 10.1053/j.jrn.2019.02.004 30956092

[B25] Rayner H. C. Larkina M. Wang M. Graham-Brown M. van der Veer S. N. Ecder T. (2017). International comparisons of prevalence, awareness, and treatment of pruritus in people on hemodialysis. Clin. J. Am. Soc. Nephrol. CJASN 12 (12), 2000–2007. 10.2215/CJN.03280317 28923831 PMC5718267

[B26] Ridefelt P. Axelsson J. Larsson A. (2012). Diurnal variability of total calcium during normal sleep and after an acute shift of sleep. Clin. Chem. laboratory Med. 50 (1), 147–151. 10.1515/cclm.2011.880 22505538

[B27] Scherer J. S. Combs S. A. Brennan F. (2017). Sleep disorders, restless legs syndrome, and uremic pruritus: diagnosis and treatment of common symptoms in dialysis patients. Am. J. kidney Dis. 69 (1), 117–128. 10.1053/j.ajkd.2016.07.031 27693261 PMC5497466

[B28] Sukul N. Karaboyas A. Csomor P. A. Schaufler T. Wen W. Menzaghi F. (2021). Self-reported pruritus and clinical, dialysis-related, and patient-reported outcomes in hemodialysis patients. Kidney Med. 3 (1), 42–53.e1. 10.1016/j.xkme.2020.08.011 33604539 PMC7873756

[B29] Sundbøll J. Szépligeti S. K. Adelborg K. Szentkúti P. Gregersen H. Sørensen H. T. (2020). Constipation and risk of cardiovascular diseases: a Danish population-based matched cohort study. BMJ open 10 (9), e037080. 10.1136/bmjopen-2020-037080 PMC7473662 32873621

[B30] Tatsuki F. Sunagawa G. A. Shi S. Susaki E. A. Yukinaga H. Perrin D. (2016). Involvement of Ca(2+)-Dependent hyperpolarization in sleep duration in mammals. Neuron 90 (1), 70–85. 10.1016/j.neuron.2016.02.032 26996081

[B31] Tian M. Song Y. Guo Y. Jiang T. (2024). Association between sleep disorders and constipation risk: a systematic review and meta-analysis. J. Clin. Neurosci. 126, 12–20. 10.1016/j.jocn.2024.05.030 38821029

[B32] Wang Q. Chen B. Sheng D. Yang J. Fu S. Wang J. (2022b). Multiomics analysis reveals aberrant metabolism and immunity linked gut microbiota with insomnia. Microbiol. Spectr. 10 (5), e0099822. 10.1128/spectrum.00998-22 36190400 PMC9602994

[B33] Wang Z. Wang Z. Lu T. Chen W. Yan W. Yuan K. (2022a). The microbiota-gut-brain axis in sleep disorders. Sleep. Med. Rev. 65, 101691. 10.1016/j.smrv.2022.101691 36099873

[B34] Weisshaar E. Weiss M. Passlick-Deetjen J. Tschulena U. Maleki K. Mettang T. (2015). Laboratory and dialysis characteristics in hemodialysis patients suffering from chronic itch--results from a representative cross-sectional study. BMC Nephrol. 16, 184. 10.1186/s12882-015-0177-3 26530958 PMC4632673

[B35] Yasuda G. Shibata K. Takizawa T. Ikeda Y. Tokita Y. Umemura S. (2002). Prevalence of constipation in continuous ambulatory peritoneal dialysis patients and comparison with hemodialysis patients. Am. J. kidney Dis. 39 (6), 1292–1299. 10.1053/ajkd.2002.33407 12046044

[B36] Yun B. Y. Sim J. Yoon J. H. Kim S. K. (2022). Association between insomnia and constipation: a multicenter three-year cross-sectional study using shift workers' health Check-up data. Saf. health A. T. work 13 (2), 240–247. 10.1016/j.shaw.2022.01.001 PMC9142359 35664914

[B37] Zhang A. Chen S. Zhu Y. Wu M. Lu B. Zhou X. (2024). Intestinal microbiome changes and mechanisms of maintenance hemodialysis patients with constipation. Front. Cell. Infect. Microbiol. 14, 1495364. 10.3389/fcimb.2024.1495364 39588509 PMC11586350

